# AccelerometerBehavior: R Package for Classifying Ungulate Behaviors Into Three States

**DOI:** 10.1002/ece3.72722

**Published:** 2025-12-18

**Authors:** Rachel A. Smiley, Seth T. Rankins, Lindsay Millward, Jack N. Gavin, Daniel P. Thompson, John A. Crouse, Peach Van Wick, Katie Anderson, Clinton W. Epps, Anna E. Jolles, Brianna R. Beechler, Rebecca L. Levine, Tayler N. LaSharr, Brittany L. Wagler, Rebekah T. Rafferty, Alyson B. Courtemanch, Tony W. Mong, Kevin L. Monteith

**Affiliations:** ^1^ Haub School of the Environment and Natural Resources University of Wyoming Laramie Wyoming USA; ^2^ Cooperative Fish and Wildlife Research Unit, Department of Zoology and Physiology University of Wyoming Laramie Wyoming USA; ^3^ Department of Fisheries, Wildlife, and Conservation Sciences Oregon State University Corvallis Oregon USA; ^4^ Alaska Department of Fish and Game Kenai Moose Research Center Soldotna Alaska USA; ^5^ Tom Thorne and Beth Williams Wildlife Research Center at Sybille, Wyoming Game and Fish Department Wheatland Wyoming USA; ^6^ School of the Environment Washington State University Pullman Washington USA; ^7^ Carlson College of Veterinary Medicine Oregon State University Corvallis Oregon USA; ^8^ Department of Integrative Biology Oregon State University Corvallis Oregon USA; ^9^ School of Computing University of Wyoming Laramie Wyoming USA; ^10^ Wyoming Game and Fish Department Jackson Wyoming USA; ^11^ Wyoming Game and Fish Department Cody Wyoming USA

**Keywords:** accelerometer, activity budgets, activity data, bighorn sheep, moose, mule deer, ungulates

## Abstract

Advances in technology provide new opportunities to study animal behavior at increasingly fine scales. GPS collars for wildlife are commonly equipped with accelerometers, which record fine‐scale movements with relatively little energy demand, yet the data remain underutilized. We paired activity data with behavioral states from direct observations and developed random forest models to classify behaviors into ‘stationary’, ‘foraging’, and ‘traveling’ states for 3 ungulate species (bighorn sheep, *Ovis canadensis* moose, *Alces alces* , and mule deer, *Odocoileus hemionus* ). Our algorithm achieved an overall classification accuracy of ≥ 87% and an area under the receiver operating curve of ≥ 0.93 for all species. The mean class error rate was 15.65% (range 4.4%–26.8%). We also developed a general ‘ungulate’ model (classification accuracy of 90% and area under the receiver operating curve of 0.95) to be applied to species lacking observation data. We developed an R package, AccelerometerBehavior, that allows users to classify ungulate behavior using our models that were validated with observation data. Unlike other packages, AccelerometerBehavior allows users to apply existing models to new activity datasets without needing their own direct observations, which can be difficult to obtain. AccelerometerBehavior facilitates the use of activity data and expands its potential for understanding ungulate behavior. Additionally, for each species, we compared activity budgets developed using AccelerometerBehavior (5‐min resolution) with those developed from Hidden‐Markov models using GPS data (1‐h resolution). Activity budgets developed using AccelerometerBehavior estimated that animals spent substantially more time stationary and less time foraging than those developed from Hidden‐Markov models and GPS data, emphasizing the need to consider the method and resolution of data when remotely assessing animal behavior. AccelerometerBehavior, which is hosted on GitHub (https://github.com/STRankins/AccelerometerBehavior), is simple and allows for the expanded use of activity data that are continuously collected on GPS collars yet are underutilized to study ungulate behavior.

## Graphical abstract

We provide an R package, AccelerometerBehavior, which facilitates the classification of acceleration data into behavioral states using field‐validated models for ungulates. Our package provides the supervised models so users do not need their own observation data for model training.

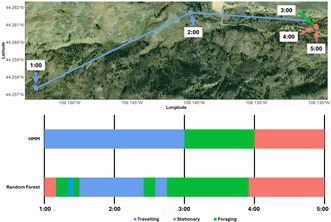

## 1 Introduction

Integrating animal behavior into ecological studies is essential to understand the habitat needs, life histories, and movements of animals (Bro‐Jørgensen et al. 2019; Williams et al. 2020). Common methods for behavioral assessments have provided valuable insights into animal ecology, though the scale or situations in which behavioral assessments are commonly conducted can present bias (Karniski et al. 2015). Direct observations of animal behavior can be limited by habitats, time of day or season, weather, or species. Increasingly, biologging technologies can provide finer‐scale and continuous insights into animal behaviors (Williams et al. 2020). Field validation, though, remains crucial for understanding the accuracy of methods to assign behaviors to remotely collected data. With the exponential growth in biologging technologies, our ability to study animal behavior and movement is rarely limited by the amount of data, but instead by our ability to connect data to behaviors at ecologically relevant scales.

GPS collars have become a cornerstone for studying space use, behavior, and informing conservation efforts for ungulates. For example, GPS data from 218 unique populations of ungulates in 11 states have allowed for the mapping of migratory corridors across the Western United States (Kauffman et al. 2025). Ungulate populations make up large portions of nation‐ and worldwide assessments of animal responses to humans that rely on GPS data (e.g., > 50% of study populations; Tucker et al. 2023; Gaynor et al. 2025), likely because they are one of the most‐commonly collared taxa. Often, studies that use GPS collars must balance the practical considerations of the GPS fix rate and the battery life of the collar, leading the vast majority of studies to use hourly to daily GPS fix rates (Kauffman et al. 2025; Tucker et al. 2023; Gaynor et al. 2025). Many GPS collars are equipped with accelerometers (usually for mortality identification), which are substantially more power‐efficient than GPS units because they do not require communication with satellites. Thus, activity data is usually collected at finer timescales (sub‐second to minute resolution) than typical GPS data (Bidder et al. 2015; Pagano and Williams 2019; Wang et al. 2015; Kauffman et al. 2025). Because of the ever‐growing number of studies that employ collars on ungulates and the increasing interest in behavior, tools that facilitate the use of activity data would provide a timely opportunity for a more in‐depth understanding of ungulate ecology.

Although accelerometers are standard on many models of GPS collars, activity data remain underutilized in behavioral studies (Wilson et al. 2015). Predicting behavior from activity data requires an algorithm derived from hours of visual observations of animals equipped with biologgers. Several R packages, such as rabc and accelerateR (Rast 2019; Yu and Klaassen 2021), provide a pipeline for cleaning, summarizing, and modeling activity data for classification into discrete behaviors. The current existing R packages, though, are wrappers for building machine learning models and require training data that is often time‐consuming, difficult, and expensive to collect. Thus far, there is not a package that provides the behavioral classification models themselves, so other users may classify behaviors without the need for training data. Providing the classification models will make behavioral classification of activity data more widely accessible.

Our goal was to use standard methods to classify ungulate behaviors using activity data paired with field observations and develop an R package to facilitate the use of activity data when field observations are unavailable. AccelerometerBehavior provides access to our classification algorithms for assigning behavioral states to segments of triaxial activity data (averaged across 5‐min intervals) from bighorn sheep ( *Ovis canadensis* ), moose ( *Alces alces* ), and mule deer ( *Odocoileus hemionus* ). We also tested a general classification model for ungulates because we expect similar movement patterns apply to basic behaviors (e.g., traveling versus stationary) across species with similar morphology. AccelerometerBehavior is easy to implement and will allow for the use of abundant, yet underutilized data to further our understanding of the behavior and ecology of ungulates.

## 2 Methods

### 2.1 Model Development

We used behavioral observations of collared ungulates from several research projects to train machine learning models that classify behavior using triaxial activity data routinely collected by GPS collars. We used data from a combination of captive and wild bighorn sheep, captive and wild mule deer, and captive moose to train our models. Use of captive animals was logistically simplest for data collection, but traveling behaviors were rare for captive animals. We therefore needed to supplement data from captive animals with data from wild animals.

### 2.2 Accelerometer and GPS Collar Specifications

For all species and study areas, we used activity data collected by accelerometers in Vertex Plus and Lite GPS collars (Vectronic Aerospace GmBH; Berlin, Germany). The accelerometer was housed on top of the collar so that it was usually positioned at the apex of the neck. All animals in the study were ≥ 2 years old. The collar bands were flexible, durable, and made from thermoplastic polyurethane membrane bonded to a base fabric.

All collars were programmed to collect activity data in Activity Mode 1 (the default mode for all Vectronic Aerospace collars), which stored an 8‐bit activity value (i.e., 0 to 255) for each of 3 axes (*X*, *Y*, and *Z*) averaged over 5‐min intervals. The following process for calculating the activity values occurred on the collar based on the standard settings. The accelerometer had a sampling rate of either 128 Hz (Vertex Plus) or 64 Hz (Vertex Lite); however, only 1 (Vertex Lite) or 4 (Vertex Plus) readings per second were used for subsequent calculations. The raw acceleration readings for the Vertex Plus (i.e., 128 Hz) were down sampled to 32 readings per second by averaging sets of 4 sequential readings. The 1st, 9th, 17th, and 25th of these 32 values were used as the 4 readings per second used for subsequent calculations. The absolute value of the difference between sequential readings was then calculated and averaged across the 5‐min window. The average of the absolute value in the difference between sequential readings was measured in units of 0.16 m/s^2^ (i.e., 0.016 g‐force). The values were then multiplied by 10 and rounded to the nearest whole number to facilitate efficient storage as an 8‐bit number with the maximum value truncated to 255 (Vectronic Aerospace, personal communications). We did not perform any processing of the activity data for our study but provide this information for users to determine if application of our method is reasonable for their collar settings.

### 2.3 Behavioral Observations for Algorithm Development

We used behavioral observations of animals from several research projects to accumulate sufficient data to develop models to classify behavior using triaxial activity data routinely collected by GPS collars. For all species, we recorded behaviors and categorized them into ‘stationary’, ‘foraging’, and ‘traveling’ behaviors. Stationary behaviors included lying, standing, and ruminating. Foraging behaviors included grazing, browsing, and feeding from troughs. Traveling behaviors included walking and running. Behaviors that did not fit into those broad categories were labeled as ‘other’ and not used for model development. ‘Other’ behaviors included alert, coughing, defecating, digging, drinking, grooming, jumping, shaking, social interactions, swimming, and urinating. By the nature of these ‘other’ behaviors, they occurred usually for short durations, unlike the behavioral states of ‘stationary’, ‘foraging’, and ‘traveling’, which animals tended to do for at least several minutes.

We used a subset of observation data collected from moose at the Kenai Moose Research Center in south‐central Alaska, USA during 2020–2021 (Kirchner et al. 2023). During 2020 and 2021, we observed 12 female moose wearing Vertex Plus GPS collars that were not supplemented with additional feed within 2.6 km^2^ enclosures containing natural vegetation.

For bighorn sheep, we collected accelerometer and observation data from 2 females wearing Vertex Plus collars at the Tom Thorne and Beth Williams Wildlife Research Center operated by the Wyoming Game and Fish Department in summer of 2022. The bighorn sheep were housed in a 0.25 ha enclosure and had access to feed troughs and naturally growing forage within their pens. To supplement data collected from the captive sheep, we used field observations of wild bighorn sheep wearing GPS collars from 2 other studies (Wyoming study and Mojave Desert study) that had ongoing research projects.

For the Wyoming project, we fitted female bighorn sheep with Vertex Lite or Vertex Plus GPS collars (Wagler et al. 2022) and conducted behavioral observations on two populations in Wyoming, USA during the summer of 2023. For the Mojave Desert project, we deployed Vertex Lite GPS collars on male and female bighorn sheep inhabiting several populations throughout the Mojave Desert, in California, USA in November 2020 (Weinstein 2025), and conducted behavioral observations throughout 2021. Field methods for the Mojave project allowed for observations of sustained movement that are rare in other observation datasets. Briefly, the project required repeated collection of fresh fecal samples from collared individuals. After a collared sheep defecated, the observers waited for the sheep to move off the defecation spot before collection; however, sometimes the sheep continued to move off in a directed manner as the fecal collector approached. The second observer continued to record the behavior of the target animal.

We collected activity and observation data from 2 female mule deer wearing Vertex Plus collars at the Washington State University Ungulate Facility in Pullman, Washington, USA during September 2023. Mule deer had access to feed troughs, naturally growing forage, and supplemental browse within their 6‐ha pens. We were unable to record enough instances of traveling (walking or running) for model training and validation and supplemented traveling behaviors inferred from GPS points from wild deer.

We leveraged data from Vertex Plus GPS collars deployed on wild mule deer in the Wyoming and Salt Ranges in Wyoming, USA in 2019 (LaSharr et al. 2023). GPS collars were programmed to collect a location once every 10 min, and we calculated the speed between sequential locations and classified steps with speeds ≥ 2.5 km/h as traveling. Traveling speeds in cervids can vary greatly, with averages of less than 1 km/h; however, walking velocities can reach 5.4 km/h (Wisdom et al. 2004; Parker et al. 1984; White and Yousef 1978). We assumed deer were traveling for the majority of the time to move at least 420 m (if the deer traveled in exact straight lines between points) within 10 min. We labeled the corresponding activity readings from the collar during these intervals as ‘traveling’ to supplement our dataset from captive deer. We used random forest models developed for moose and bighorn sheep (details in the next section) to evaluate support for labeling these segments as traveling.

### 2.4 Random Forest Model Development

We used Breiman's random forest models for classification, within the randomForest (Liaw and Wiener 2002) package in R, to classify behavior (stationary, foraging, and traveling) from 5‐min blocks of averaged triaxial activity data (*X*, *Y*, and *Z* values). Random forest models are a class of supervised machine learning algorithms. Specifically, random forest classification models use a bootstrapped sample of labeled training data to create a prediction tree (Liaw and Wiener 2002). The final random forest classification model is a majority vote aggregate of all the independent prediction trees grown from each bootstrapped sample of data (Breiman 2001; Liaw and Wiener 2002). We implemented separate models for bighorn sheep, mule deer, and moose. For mule deer and bighorn sheep, we used only 5‐min segments that were a single behavior based on our direct observations. Our sample size for moose data necessitated that we use 5‐min segments that were a single behavior for ≥ 3 min to have enough data for modeling purposes. We deemed this acceptable, because our moose model was able to perform satisfactorily (i.e., ≥ 0.9 AUC) even when activity data from time periods with heterogeneous behavior were included. We grew 1001 prediction trees for each model. Using an odd number of prediction trees ensured that models would converge in the rare instances that we had a tie at a node. The number of variables that were randomly sampled as candidate parameters for each node was left at the default, which is the square root of the total number of possible parameters (Liaw and Wiener 2002). Because our data had imbalanced sample sizes between categories, we drew equally sized bootstrapped samples of data from each behavior category for every tree grown (Boulesteix et al. 2012; Liaw and Wiener 2002). The number of samples drawn was the number of observations in the behavior category with the smallest sample size.

We used a leave‐one‐out cross‐validation with unique animals serving as the sampling unit to test the performance of our models. We used a combination of overall model accuracy and the area under the operating receiver curve (AUC; Hand and Till 2001) to assess model fit (Probst and Boulesteix 2018). Once we achieved ≥ 0.9 AUC, we created species‐specific classification models using the full data set. We then used the same process to create a general ungulate model using all 5‐min segments that were a single behavior from all 3 species. We developed the *AccelerometerBehavior()* function to predict behaviors, housed in the AccelerometerBehavior package for Program R, from our prediction models that were trained on the full datasets. The AccelerometerBehavior package is hosted on GitHub (https://github.com/STRankins/AccelerometerBehavior).

### 2.5 Comparisons of Methods to Classify Behaviors

AccelerometerBehavior is simple, making it widely accessible for researchers and managers. For context about the use of our method compared to a standard method for remotely classifying behaviors, we present a case study for each species (i.e., bighorn sheep, moose, and mule deer) to highlight the differences between AccerlerometerBehavior and Hidden Markov models (HMMs), which use non‐continuous GPS location data. Our GPS and activity datasets are collected at different temporal resolutions. Thus, this comparison only serves to provide context, not to compare the accuracy of methods, which would require several hours of continuous observation of animals. HMMs use relatively long step lengths to classify traveling behaviors, which inherently would make continuous observation difficult. Further, because we prioritized using all our observation data for model training, we were unable to compare the accuracy of methods with a separate dataset.

We leveraged existing location and activity data from GPS collars deployed in northwestern Wyoming on wild mule deer, bighorn sheep, and moose to compare activity budgets generated using our random forest models to those generated using HMMs. Wild ungulates throughout western Wyoming were collared for other research objectives and details of the collaring procedures are outlined for each population elsewhere (Wyoming and Salt ranges mule deer from 2019 to 2023; LaSharr et al. 2023; Whiskey Mountain and Gros Ventre ranges bighorn sheep from 2019 to 2023; Wagler et al. 2022; Meeteetse study moose from 2020 to 2022; Levine et al. 2022). All GPS collars were either Vertex Plus or Vertex Lite collars that were programmed to collect hourly GPS locations and had an onboard accelerometer programmed to Activity Mode 1. Only adult females were used from each study because of data availability. For each individual, we randomly selected 2 weeks to compare activity budgets derived from our random forest models to those generated using HMMs.

We developed three‐state HMMs to predict stationary, foraging, and traveling behaviors using the *fitHMM()* function in the momentuHMM package (McClintock and Michelot 2018). We developed separate HMMs derived from only sequential GPS location data (i.e., step length and turning angle) and models that used both sequential GPS location and triaxial activity data at hourly intervals for each species. We specified a gamma distribution for the step length, a wrapped‐Cauchy distribution, and gamma distributions for each activity value for the state‐dependent probability distributions within the HMMs. For HMMs that used both GPS and triaxial activity data, we averaged the activity values across each hour to match the time intervals of the GPS data. We then used the 3 values of hourly activity (*X*, *Y*, and *Z*‐axes) as data streams within the HMMs. We ran 50 iterations of each model with different starting parameters that were drawn from specified distributions and selected the best model based on Akaike's information criterion (AIC; Burnham and Anderson 2004). Finally, we compared HMMs using GPS data only to those that also included activity values and used the model with the lower AIC value.

Using the final HMM models for each species, we predicted the most probable state sequences for each animal using the *viterbi()* function in the R package momentuHMM (McClintock and Michelot 2018) and then calculated the proportion of time individuals were in each behavioral state. We then generated activity budgets using A*ccelerometerBehavior()* using activity data from the same 2‐week window for the same individuals, so we could directly compare activity budgets generated by the two methods. We calculated the proportion of time animals were in each behavioral state as determined by the random forest models contained in AccelerometerBehavior. We randomly selected 25 animals for each species for budget comparisons. Lastly, we compared the proportion of time spent in each state as determined by the two methods using paired *t*‐tests and reported the mean differences.

### 2.6 Ethics Statement

All animal related activities were approved by and followed Institutional Animal Care and Use Committee (IACUC) protocols (captive mule deer: Washington State University's IACUC protocol #7230; captive moose: Alaska Department of Fish and Game Division of Wildlife Conservation Animal Care and Use Committee protocols 0086‐2020‐40 and 0086‐2021‐55; captive bighorn sheep: Tom Thorne and Beth Williams Wildlife Research Center operated by the Wyoming Game and Fish Department IACUC protocol #16‐03; wild desert bighorn sheep: California Department of Fish and Wildlife‐Wildlife Health Lab, National Park Service IACUC permit CA_MOJA_Beechler.Epps_DBHS_2020, and Oregon State University IACUC permit IACUC‐2019‐0017. Desert bighorn research was conducted under National Park Service permit MOJA‐2020‐SCI‐0031; wild Rocky Mountain bighorn sheep: University of Wyoming IACUC protocol #20180305KM00296‐03; wild moose: University of Wyoming IACUC protocols 20200305KM00412‐01 and 20220111KM00532‐01; wild mule deer: University of Wyoming IACUC protocols 20170215KM00260 and 20200305KM00412). When possible, we used existing data for testing and validation instead of repeating efforts.

## 3 Results

We used 175 h of direct observations of 63 unique animals to develop algorithms to classify behavioral states from accelerometer data (Table 1). To determine if there was support for using GPS data with movement speeds > 2.5 km/h for mule deer traveling states, we evaluated how the models for the other species classified the data points that we labeled traveling for mule deer. The random forest model for moose (another cervid) labeled 91% of the datapoints as traveling, and the model for bighorn sheep (a bovid) labeled 59% of the datapoints as traveling. These datapoints for traveling, which were inferred as traveling from GPS data instead of direct observations, represent 1.7% of all of the data used for algorithm development.

**TABLE 1:** Data sources and sample sizes used for behavioral classification algorithm development and for comparing activity budgets calculated with Hidden‐Markov models to those calculated using accelerometer data.

Species	Data source	Model use and sample size	
		Algorithm development	HMM‐accelerometer comparison
Bighorn sheep	Tom Thorne and Beth Williams Wildlife Research Center (captive)	15.0 h	—
Bighorn sheep	Mojave Desert Study (wild)	48.4 h	—
Bighorn sheep	Western Wyoming Study (wild)	6.2 h	50 weeks
Moose	Kenai Moose Research Center (captive)	83.0 h	—
Moose	Meeteetse Study (wild)	—	50 weeks
Mule deer	Washington State Ungulate Facility (captive)	20.6 h	—
Mule deer	Wyoming Range Study (wild)	1.8 h (traveling only)	50 weeks

### 3.1 Random Forest Models

Random forest models for each species had overall classification accuracies between 87% and 95%, AUC values between 0.93 and 0.99, and out‐of‐bag error rates between 6.6% and 13.4% (Tables 2 and 3). Prediction accuracy from the combined ungulate model applied to each species was 88% for bighorn sheep, 94% for mule deer, and 90% for moose. Model accuracy on the training datasets for our moose, bighorn sheep, mule deer, and ungulate models was 91%, 89%, 97%, and 92%, respectively. In comparison, model accuracy on the testing datasets for our moose, bighorn sheep, mule deer, and ungulate models was 90%, 87%, 93%, and 90%, respectively, indicating that we did not overfit our models.

**TABLE 2:** Number of 5‐min intervals, overall classification accuracy, AUC, out‐of‐bag error rate, and class error rates for random forest prediction models for classifying ungulate behavior from 5‐min segments of triaxial activity data based on means of animals with ≥ 30 observations from leave‐one‐out cross‐validation using unique animals as the sampling unit.

Model	*n*	Classification accuracy (%)	AUC	Out‐of‐bag error rate (%)	Class error rate (%)		
					Foraging	Stationary	Traveling
Bighorn sheep	835	87	0.93	13.4	25.0	7.8	24.4
Moose	996	90	0.96	10.2	8.2	13.5	15.7
Mule deer	268	93	0.98	7.3	14.6	4.4	15.9
Ungulate	1327	90	0.95	11.9	25.5	6.0	26.8

**TABLE 3:** Number of 5‐min intervals, overall classification accuracy, AUC, out‐of‐bag error rate, and class error rates for random forest prediction models for classifying ungulate behavior from 5‐min segments of triaxial activity data based on medians of leave‐one‐out cross‐validation using unique animals as the sampling unit.

Model	*n*	Classification accuracy (%)	AUC	Out‐of‐bag error rate (%)	Class error rate (%)		
					Foraging	Stationary	Traveling
Bighorn sheep	835	91	0.99	13.2	24.5	7.8	24.5
Moose	996	93	0.98	9.7	7.8	13.0	15.0
Mule deer	268	95	0.98	6.6	14.7	4.7	18.2
Ungulate	1327	94	0.99	11.9	25.4	6.2	27.4

### 3.2 Activity Budget Comparisons

**FIGURE 1:**
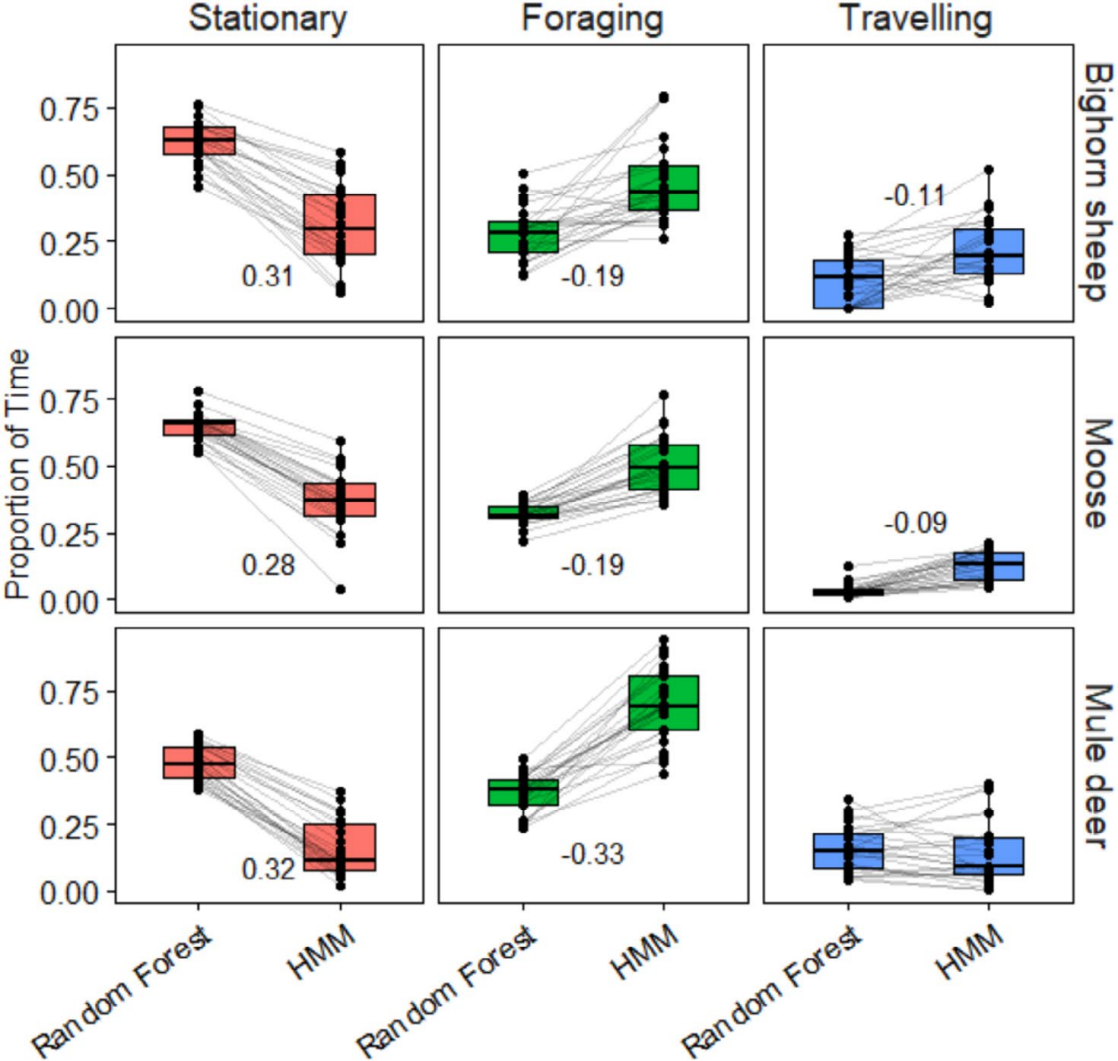
Activity budgets generated for bighorn sheep, moose, and mule deer (*n* = 25 per species) during a 2‐week window randomly selected throughout the year. We used activity data recorded every 5 min for Random Forest models (housed in the AccelerometerBehavior package) and GPS location data recorded once per hour for Hidden Markov Models (HMMs) for all 3 species. Numbers within the plots indicate the mean difference between the proportion of time spent in each state if the difference was significant (*p* < 0.001). There was no difference in the predicted proportion of time spent traveling between the methods for mule deer.

**FIGURE 2:**
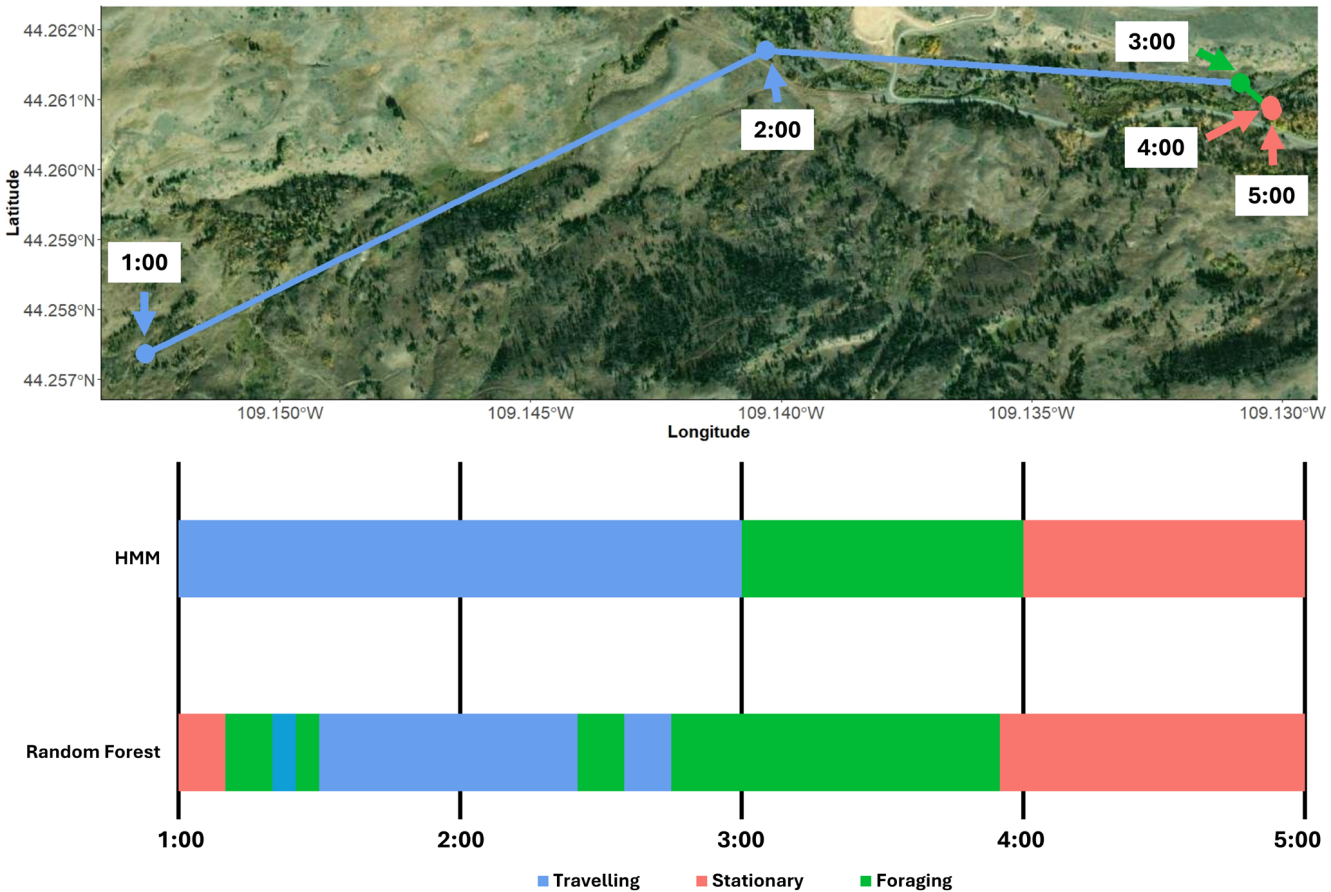
An example of the difference between behaviors of a moose as derived from HMMs (colored lines and points and top bar) using hourly GPS data and from the random forest model used by the *AccelerometerBehavior()* function using triaxial activity data at a 5 min resolution (bottom bar). Black vertical lines denote hour marks.

We also generated activity budgets for wild bighorn sheep, moose, and mule deer in Western Wyoming (25 individuals for each species; Table 1), and compared them to activity budgets developed using HMMs, which are a standard, yet usually unvalidated, method of classifying ungulate behaviors (Figures 1 and 2). We classified behaviors using HMMs with GPS data, HMMs with GPS and activity data, and Random Forest models contained in AccelerometerBehavior. For all three species, the HMMs with GPS data only outperformed HMMs with GPS and activity data (bighorn sheep ΔAIC: 302762.80, moose ΔAIC: 154585.20, mule deer ΔAIC: 712632.8).

For all three species, activity budgets generated using the *AccelerometerBehavior()* function predicted less time foraging and more time stationary compared with activity budgets generated with HMMs (Table 4, Figure 1), indicating that activity budgets are sensitive to the temporal resolution of the data and method used. Specifically, activity budgets generated using the *AccelerometerBehavior()* function predicted approximately 20 percentage points less time spent foraging for bighorn sheep and moose and 32 percentage points less time spent foraging for mule deer than the budgets generated using HMMs (Figure 1, Table 4). Activity budgets generated with AccelerometerBehavior predicted that all three species spend more time stationary than foraging, whereas activity budgets generated from HMMs predicted that all three species spend more time foraging than stationary.

**TABLE 4:** Comparison of activity budgets generated with random forest models that used 5‐min averaged triaxial activity data and budgets generated with Hidden Markov Models that used hourly GPS data. Degrees of freedom for each paired *t*‐test was 24.

Species	Behavior	t‐value	*p*
Bighorn sheep	Stationary	13.54	< 0.001
	Foraging	−5.45	< 0.001
	Traveling	−4.21	< 0.001
Moose	Stationary	18.43	< 0.001
	Foraging	−10.81	< 0.001
	Traveling	−10.09	< 0.001
Mule deer	Stationary	25.53	< 0.001
	Foraging	−12.63	< 0.001
	Traveling	0.71	0.48

### 3.3 AccelerometerBehavior R Package Overview

The AccelerometerBehavior package is an R package hosted on GitHub for classifying behavioral states of ungulates using 5‐min segments of activity data. Our package is unique because it allows users to determine activity states from 5‐min segments of triaxial, 8‐bit activity data without the need for corresponding observation data. Users can input a dataframe with a minimum of 3 columns, which are the *X*, *Y*, and *Z* activity data obtained from Vertex Plus or Lite GPS collars or similarly configured accelerometer mounted to a collar. The AccelerometerBehavior package contains the *AccelerometerBehavior()* function. Users must specify 5 arguments: the species argument (“mule deer”, “bighorn sheep”, “moose”, or “ungulate”), the *X*, Y, and *Z* arguments, and the name of the data frame containing the data. The output is the original data frame with an additional column named ‘behavior’ with the most probable behavioral state (i.e., stationary, foraging, traveling) as determined by the random forest model for the specified species.

For users with triaxial acceleration data (the raw values of acceleration over time), rather than triaxial activity data (a summary metric of variation in the acceleration data—the standard output for some GPS collars), we provide the function *FirstDiff2Act()* to aid in conversion of units. The *FirstDiff2Act()* function requires 2 arguments. The first argument should be a numeric value or vector of numeric values of the absolute value of the average first difference in acceleration for a 5‐min period of time, which may be calculated using existing base R functions. The second argument should be the units, either “g” or “m/s^2^” (i.e., g‐force or m/s^2^), in the input data that was measured. The output of the function is the data after it has been converted to the 8‐bit activity data, which the *AccelerometerBehavior()* function requires.

The AccelerometerBehavior package is licensed under a GPL ≥ 2.0 license. The AccelerometerBehavior package has just two long‐standing packages as dependencies (stats and randomForest packages), meaning our package is relatively robust to future version changes and will have a long lifecycle.

### 3.4 Example Workflow

# Load package

*library(AccelerometerBehavior)*

*#* The absolute value of the averaged first difference in acceleration for a 5‐minute time period

# can be converted to the required 8‐bit activity data using the *FirstDiff2Act()* function. We

# expect this will be unnecessary for most users because the activity value is usually calculated

# on the collar.

# Simulate data set

*x <‐ runif(10, 0, 1)*

# Convert the data to the corresponding 8‐bit activity data

*FirstDiff2Act(x, units = “g”)*

*#* Simulate dataset, activity values must range from 0 to 255

*ActivityX <‐ round(rnbinom(400, size = 1, mu = 20), 0)*

*ActivityX[ActivityX > 255] <‐ 255*

*ActivityY <‐ round(rnbinom(400, size = 1, mu = 20), 0)*

*ActivityY[ActivityY > 255] <‐ 255*

*ActivityZ <‐ round(rnbinom(400, size = 1, mu = 20), 0)*

*ActivityZ[ActivityZ > 255] <‐ 255*

*df <‐ data.frame(ActivityX, ActivityY, ActivityZ)*

*#* Run *AccelerometerBehavior()* function, which adds a 'behavior' column to the dataframe

*AccelerometerBehavior(data = df, species = “moose”, x = “ActivityX”, y = “ActivityY”, z = “ActivityZ”)*

## 4 Discussion

Biologging technologies have the potential to rapidly advance our understanding of how animals interact with their environments (Beltran et al. 2024). Accelerometers support the efficient classification of behaviors (Rast 2019; Yu and Klaassen 2021), which can complement the spatial data collected with GPS collars. We developed random forest models that classified behavioral states from activity data paired with direct observations of 3 ungulate species (bighorn sheep, moose, and mule deer). We housed the random forest models in AccelerometerBehavior, an R package that allows users to implement the models to classify behaviors with their own activity datasets. We followed the standard workflow and methodology of classifying behaviors from triaxial activity data using machine learning algorithms (Brandes et al. 2021; Kirchner et al. 2023; Nathan et al. 2012) and made our classification system publicly available. To our knowledge, AccelerometerBehavior is the first package that provides pre‐existing models to classify behavioral states from activity data, as opposed to existing packages, which support users in developing models from paired observation‐activity data that the user must provide. By creating a simple function for sharing and implementing predictive models to classify ungulate behavior, we will help ecologists unlock the full potential of animal‐borne accelerometers, further integrating behavior and ecology.

We collectively spent 175 h observing animals to train the models within AccelerometerBehavior, which does not include the additional hours of planning, training, travel to and from study sites, time spent searching for collared animals to observe, data entry, data cleaning, or financial costs involved with behavioral observations. By providing our supervised models, we hope to save other researchers from repeating these efforts unnecessarily. Although it may be ideal to validate and build species or system‐specific models, sometimes it is impractical or impossible to conduct intensive field observations. In addition to models for bighorn sheep, moose, and mule deer, we provide a general ‘ungulate’ option in our package for use in these situations. Our ungulate model may be used for classifying behavioral states of rare or hard‐to‐observe ungulate species for which paired observation and activity data are unavailable, although we acknowledge that classification accuracy will be reduced compared to species‐specific models. Our general ungulate model maintained an overall classification accuracy of 88%, while providing a field‐validated method of assigning behavioral states with biological relevance (i.e., foraging rather than encamped) to readily available activity data. Depending on the goals of the study and the ecology of the study species, we encourage researchers to carefully consider the methods they use for classifying behaviors (Bennison et al. 2018; Buderman et al. 2021) because differences in results can have cascading implications for management and conservation efforts. Technology is rapidly improving our ability to learn about animal behavior, and accelerometers can provide near continuous activity budgets for hard to observe animals.

The questions we can ask and the inferences we can make with new technologies hinge on the temporal resolution at which we collect data. Animal behavior operates at a sub‐minute level (Marshall et al. 2021; Mazzucato 2022), and we detected differences in activity budgets in wild animals depending on the temporal resolution of data and method of classification used (Figures 1 and 2). Practical limitations of GPS units (e.g., weight, size, and lifespan of the battery) result in GPS data that are often collected at the scale of hours or days. Accelerometers have substantially lower energy requirements than GPS units and, therefore, can efficiently collect data at temporal resolutions that better match the temporal scale of animal behavior. For bighorn sheep, mule deer, and moose, there were substantial differences in activity budgets generated using 5‐min activity data and hourly GPS data (Figures 1 and 2). We did not explicitly test the accuracy of HMMs, which are usually unsupervised, but instead our comparison served to provide context for differences in activity budgets generated with activity data compared to coarser‐scale GPS data. Regardless of the method used, the interpretation and assessment of animal behavior should match the temporal resolution of the data (Leos‐Barajas et al. 2017).

## 5 Conclusions

AcceleromerBehavior provides users with an accurate yet easy method to create activity budgets for three ungulate species with international distributions–bighorn sheep, moose, and mule deer. Our R package provides a field‐validated method to classify behavior without the need to spend time and resources collecting direct observation data for model training. Because our package only uses *X*, *Y*, and *Z* activity values that were averaged over 5 min, users can implement AccelerometerBehavior with standard collar settings without sacrificing battery life, storage space for spatial data, or dealing with especially large and unwieldy datasets. Our R package will allow researchers the opportunity to easily collect behavioral data at large temporal and spatial resolutions, which may yield a pathway to better integrate animal behavior within other disciplines of ecology. AccelerometerBehavior has potential for growth, and we welcome contributions to the package through paired activity and behavioral data of other species.

## Author Contributions

**Rachel A. Smiley:** conceptualization (equal), data curation (equal), formal analysis (equal), investigation (equal), methodology (equal), project administration (equal), visualization (equal), writing – original draft (equal), writing – review and editing (equal). **Seth T. Rankins:** conceptualization (equal), data curation (equal), formal analysis (equal), investigation (equal), methodology (equal), project administration (equal), software (equal), validation (equal), visualization (equal), writing – original draft (equal), writing – review and editing (equal). **Lindsay Millward:** data curation (equal), resources (equal), writing – original draft (supporting), writing – review and editing (lead). **Jack N. Gavin:** data curation (lead), writing – review and editing (lead). **Daniel P. Thompson:** data curation (lead), writing – review and editing (lead). **John A. Crouse:** data curation (lead), writing – review and editing (lead). **Peach Van Wick:** data curation (lead), writing – review and editing (lead). **Katie Anderson:** data curation (supporting), writing – review and editing (lead). **Clinton W. Epps:** data curation (lead), funding acquisition (lead), writing – review and editing (lead). **Anna E. Jolles:** data curation (lead), funding acquisition (lead), writing – review and editing (lead). **Brianna R. Beechler:** data curation (lead), funding acquisition (lead), writing – review and editing (lead). **Rebecca L. Levine:** data curation (lead), writing – review and editing (lead). **Tayler N. LaSharr:** data curation (lead), writing – review and editing (lead). **Brittany L. Wagler:** data curation (lead), writing – review and editing (lead). **Rebekah T. Rafferty:** data curation (lead), writing – review and editing (lead). **Alyson B. Courtemanch:** data curation (supporting), funding acquisition (supporting), writing – review and editing (lead). **Tony W. Mong:** data curation (supporting), funding acquisition (supporting), writing – review and editing (lead). **Kevin L. Monteith:** data curation (supporting), funding acquisition (lead), writing – review and editing (supporting).

## Funding

This work was supported by the Wyoming Wildlife Livestock Disease Research Partnership, Knobloch Family Foundation, Wyoming Wildlife and Natural Resource Trust, Wyoming Governors Big Game License Coalition, Wild Sheep Foundation, Muley Fanatic Foundation, Boone and Crockett Club, Wyoming Wild Sheep Foundation, UW‐NPS Research Station, A. Young, Bowhunters of Wyoming, Wyoming Animal Damage Management Board, Pope and Young Club, Wyoming Outfitters and Guides Association, U.S. Fish and Wildlife Service, U.S. Forest Service, Wyoming Game and Fish Department, National Institute of Food and Agriculture, McIntire‐Stennis Project (WNP00848), Washington State University, U.S. Bureau of Land Management, NSF‐NIH‐NIFA Ecology and Evolution of Infectious Disease (1911994), M. and C. Rumsey, UK Biotechnology and Biological Sciences Research Council (BB/T011416/1), J. Nielson, M. Newhouse.

## Conflicts of Interest

The authors declare no conflicts of interest.

## Data Availability

All data and code are available through Zenodo (https://doi.org/10.5281/zenodo.17834808).
